# Electronic Band Alignment at Complex Oxide Interfaces Measured by Scanning Photocurrent Microscopy

**DOI:** 10.1038/s41598-017-04265-9

**Published:** 2017-06-19

**Authors:** J. H. Yoon, H. J. Jung, J. T. Hong, Ji-Yong Park, Soonil Lee, S. W. Lee, Y. H. Ahn

**Affiliations:** Department of Physics and Department of Energy Systems Research, Ajou University, Suwon, 16499 Korea

## Abstract

The band alignment at an Al_2_O_3_/SrTiO_3_ heterointerface forming a two-dimensional electron gas (2DEG) was investigated using scanning photocurrent microscopy (SPCM) in an electrolyte-gated environment. We used a focused UV laser source for above-the-bandgap illumination on the SrTiO_3_ layer, creating electron-hole pairs that contributed to the photocurrent through migration towards the metal electrodes. The polarity of the SPCM signals of a bare SrTiO_3_ device shows typical p-type behavior at zero gate bias, in which the photogenerated electrons are collected by the electrodes. In contrast, the SPCM polarity of 2DEG device indicates that the hole carriers were collected by the metal electrodes. Careful transport measurements revealed that the gate-dependent conductance of the 2DEG devices exhibits n-type switching behavior. More importantly, the SPCM signals in 2DEG devices demonstrated very unique gate-responses that cannot be found in conventional semiconducting devices, based on which we were able to perform detailed investigation into the electronic band alignment of the 2DEG devices and obtain the valence band offset at the heterointerface.

## Introduction

Heterointerfaces between complex oxide insulators have unique properties that are not observed in conventional electronic devices^1, 2^. In particular, high density two dimensional electron gases (2DEGs) can be generated at the epitaxial interface of two perovskite-insulators, e.g., between LaAlO_3_ (LAO) and SrTiO_3_ (STO) crystals^3–6^. As devices are scaled down to atomic dimensions, transistors will require extremely high charge densities. Silicon devices typically have two-dimensional electron gas densities of around 10^12^ cm^−2^; however, the 2DEG at the interface of oxide heterostructures such as LAO/STO or Al_2_O_3_/STO has charge densities as high as >10^13^ cm^−2^ at the nanometer scale. Therefore, oxide heterostructures have emerged as a novel platform in the development of advanced field-effect transistors (FETs) for high-speed electronics and sensors^6–14^.

The extraordinarily high carrier density at the heterostructures can be interpreted through the polar catastrophe mechanism^15–18^, where the metallic channel is created above the critical thickness of the LAO film. Recently it has been reported that a 2DEG can be created by growing amorphous LAO or Al_2_O_3_ films on the STO substrate^19–22^, in which case the polar catastrophe mechanism does not apply. The formation of the 2DEG when using the amorphous oxides has been attributed to the creation of oxygen vacancies (VO) on the STO surface, inducing the creation of high-density free electrons^20–24^. These amorphous LAO and Al_2_O_3_ layers have been grown using atomic layer deposition (ALD)^20, 23^, which is widely used to grow functional films of high quality, providing mass production compatibility^25^.

In order to understand the underlying physical implications and to optimize these unique devices for practical applications, it is important to obtain detailed information on the electronic band structures, especially at the heterointerfaces, with nanoscale spatial resolution. Over the past decade, scanning photocurrent microscopy (SPCM) has been successfully used to study the electrical and photoelectrical properties of various nanoscale devices^26–30^. Information obtained from SPCM includes the band structure of the metallic contacts, junctions, and defects. On the other hand, SPCM has not been used to study the localized characteristics of complex oxide heterostructures, primarily because of the large bandgap of the materials involved.

In this work, we investigated the band alignment of 2DEG systems using SPCM under illumination with a focused UV laser whose energy is above the band-gap of the STO. In this configuration, electron-hole pairs are generated in the STO layer and are then collected by the nearby metal electrodes. This enabled us to study the band alignment of 2DEG systems in conjunction with their gate-dependent photocurrent response.

## Results and Discussion

### Sample preparation and experimental setup

**Figure 1:**
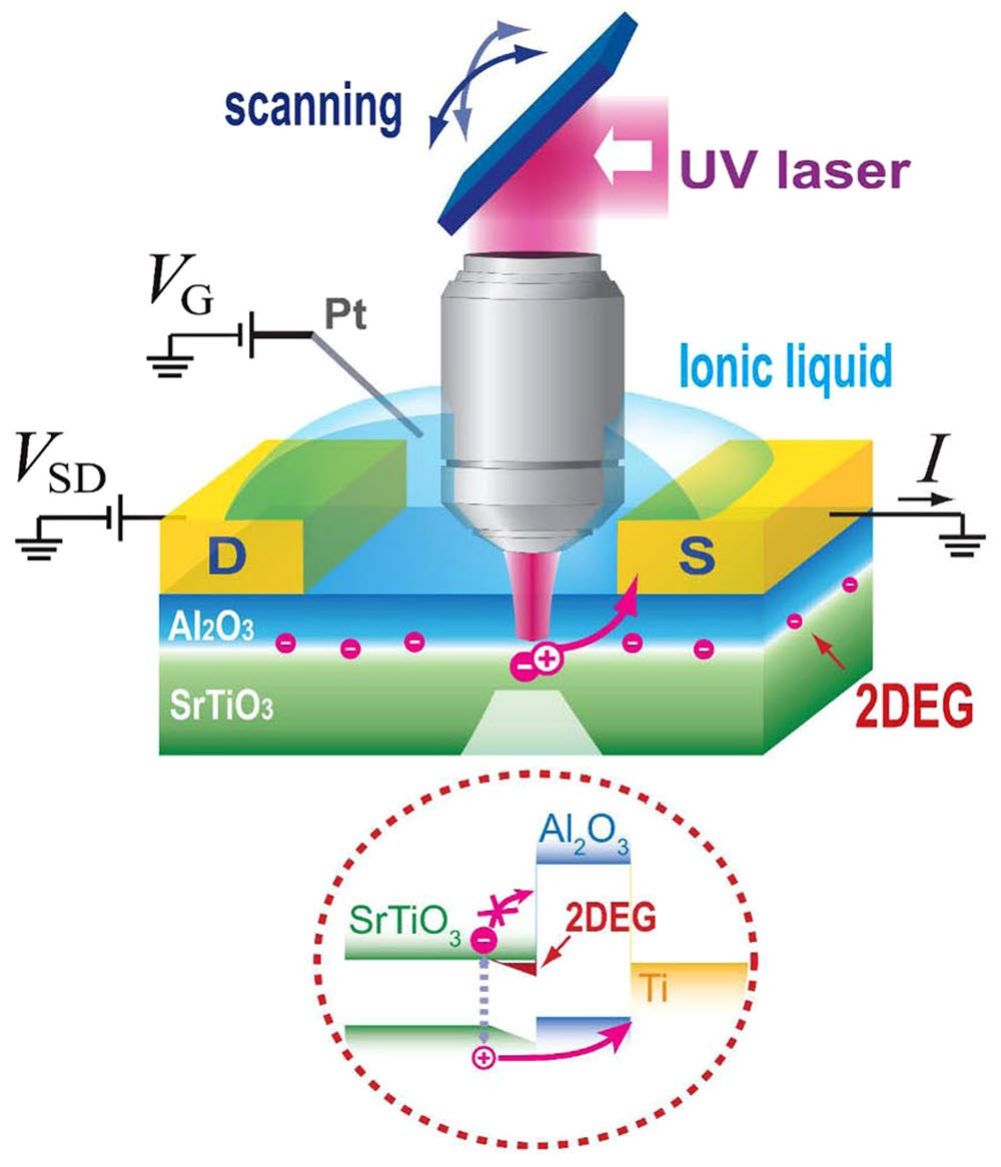
Schematic of SPCM measurement on a 2DEG device. We used a UV (355 nm) laser for the above-band-gap illumination on STO layer.

We fabricated FET devices with a 2DEG layer formed at the Al_2_O_3_/STO interface. The amorphous Al_2_O_3_ films (with 5 nm thickness) were grown on a TiO_2_-terminated (001) STO crystalline substrate at 300 °C using the ALD technique. The metal electrodes were defined using conventional photolithography, followed by metal evaporation (Ti/Pt). The sheet resistance of the conducting heterointerfaces is ~40 MΩ/sq. with the typical Hall electron mobility of 5 cm^2^/Vs at room temperature^20^. For efficient gating, we used the ionic liquid gating method with a Pt electrode submerged in ionic liquid^31^. As schematically shown in Fig. 1, we scanned the focused UV laser (2.8 mW) over the samples, while simultaneously monitoring the photo-induced signals as a function of the laser’s position^26–29^. To address the device operation in an electrolyte environment, we used a water-immersion type objective lens (Supplementary Information S1)^32^. Because the energy of the UV laser (3.5 eV) is higher than the STO bandgap (3.2 eV)^3^, it induces electron and hole carriers in the STO region. These carriers migrate towards the nearby electrodes while being influenced by the electronic band alignment at the interface.

### SPCM imaging on bare SrTiO_3_ devices

**Figure 2:**
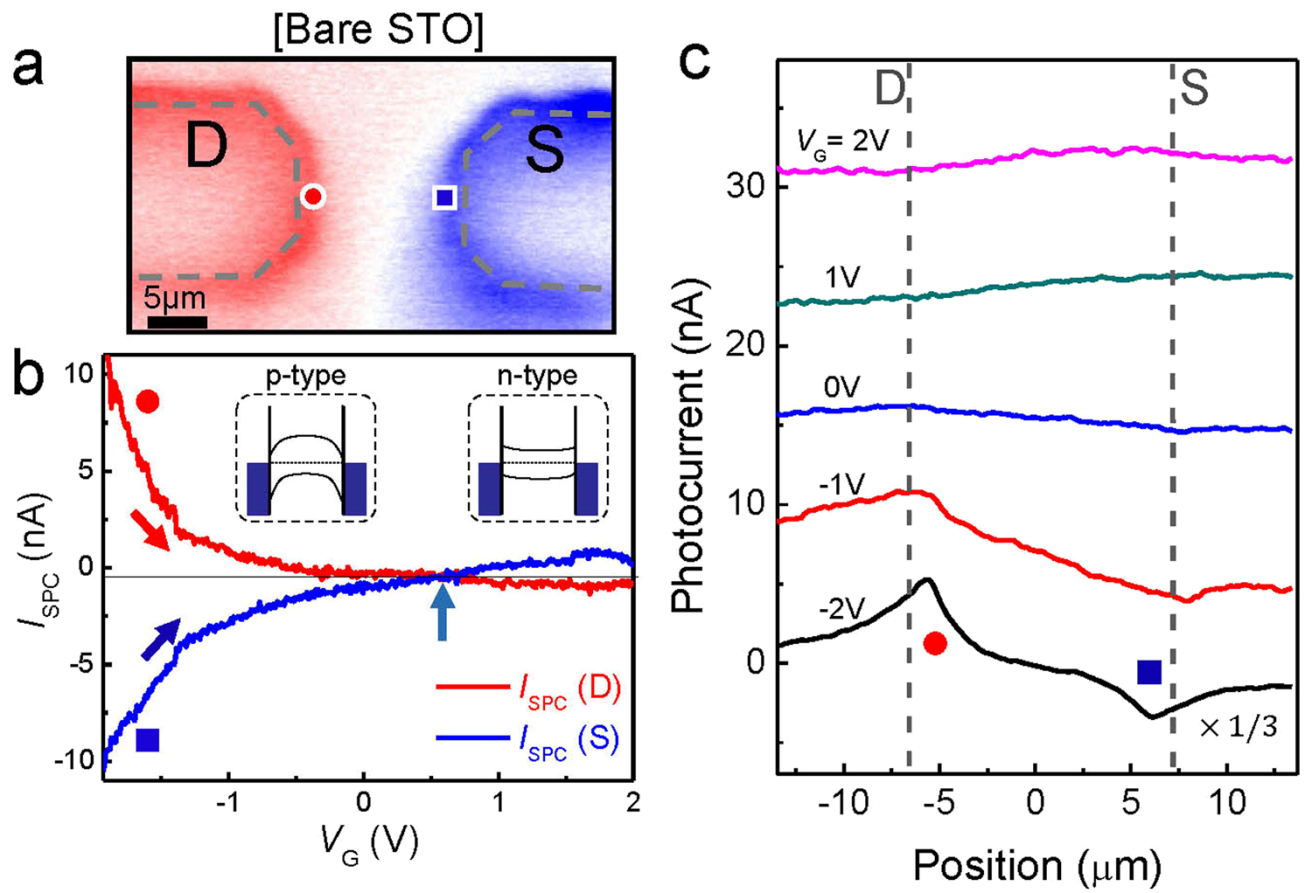
(**a**) SPCM of a bare STO device at *V*_SD_ = *V*_G_ = 0 (**b**) Photocurrent as a function of *V*_G_ near the drain (red) and source (blue) electrodes. (inset) Schematic band diagrams of STO device for p-type (left), and n-type (right) operation. (**c**) Photocurrent profile as a function of position for various *V*_G_’s. The curves are offset for clarity.

We begin with SPCM measurements on a bare STO device, i.e., without the Al_2_O_3_ layer on top. A representative SPCM image is shown in Fig. 2a submerged in the ionic liquid solution. The source-drain voltage (*V*_SD_) and the ionic liquid gate voltage (*V*_G_) were fixed at zero (i.e., at *V*_G_ = *V*_SD_ = 0 V). Although the STO substrate is an insulator, we were able to observe strong SPCM signals near the metal contacts (*I*_SPC_) at a very low laser power of 10 μW (Supplementary Information S2). We obtained a positive and negative photocurrent near the drain and source electrodes, respectively. This *I*_SPC_ polarity can be interpreted as p-type band bending near the metal electrode, in which the electronic potential becomes higher toward the middle of the conduction channel, as depicted by the band diagram in the inset of Fig. 2b. This implies that it is the photogenerated electron carriers that are collected by the metal electrodes, as in the case of previous results on various semiconducting devices with p-type bending near the electrode^27–30^.

Interestingly, the SPCM varied dramatically with electrolyte gate voltage. In Fig. 2b, we plotted *I*_SPC_ signals as a function of *V*_G_ near the drain and the source electrodes, as depicted by a red circle and a blue square, respectively. As we increased *V*_G_ from −2 V towards positive values, *I*_SPC_ decreased significantly for both the drain and source electrodes. Furthermore, polarity switching behavior (i.e., the sign of *I*_SPC_ changed from the positive to negative for the drain electrode) was observed at *V*_G_ ~ 0.6 V, which is the gate voltage where the bending behavior changed from p-type to n-type. In addition, the photocurrent profiles measured along the conduction channel are shown in Fig. 2c. Consistent with those in Fig. 2b, the *I*_SPC_ signals are pronounced at large negative *V*_G_ values (e.g. *V*_G_ = −2 V), with polarity comparable to the p-type behaviors. They decrease with increasing *V*_G_ and the polarity switching behavior is found for the results with *V*_G_ = 1 V and 2 V.

Our observation of p-type band bending at *V*_G_ = 0 V is consistent with the prediction derived from material parameters such as the work function of Ti (*Ф*_B_ ~ 4.33 eV) and the electron affinity of STO (*χ*_e_ = 3.9 eV) (Supplementary Information S3). However, our understanding of the gate-dependence of the SPCM signals with respect to the electronic band alignment is limited, because we could not observed the gate-response in the DC conductance measurement for the bare-STO devices that act as insulators in the absence of UV illumination. Conversely, the electronic band structure of the Al_2_O_3_/STO heterointerface can be studied in detail by using the SPCM signals at the metal contacts in conjunction with gate-dependent transport measurements^27–30^.

### SPCM imaging on 2DEG devices

**Figure 3:**
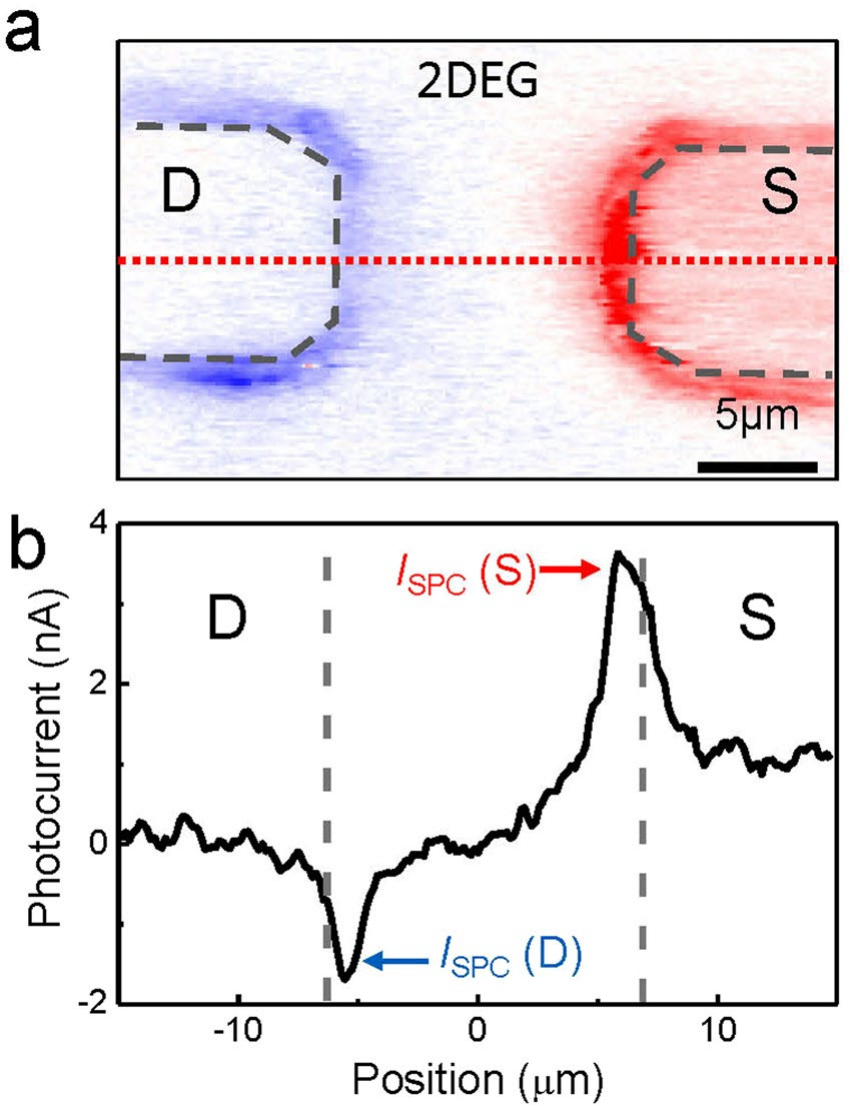
(**a**) A SPCM image of 2DEG device taken at *V*_SD_ = *V*_G_ = 0. Red (blue) indicates positive (negative) current. (**b**) Photocurrent profile as a function of position, extracted from (**a**) along the red dotted line.

Surprisingly, the SPCM signals for the 2DEG device exhibit a polarity that is opposite to that of the bare STO case. A representative SPCM image is shown in Fig. 3a in the ionic liquid solution (at *V*_G_ = *V*_SD_ = 0 V). Photocurrent profiles measured along the dotted line is shown in Fig. 3b. The *I*_SPC_ were much lower in 2DEG than in the bare STO devices (by about two orders of magnitude), and hence, we used a higher laser power (2.8 mW). This is due to the Al_2_O_3_ layer that blocks the photogenerated carriers, which reduces the photocurrent signals captured by the metal electrodes. At zero gate bias, we generally observed a negative photocurrent near the drain electrode and a positive current near the source electrode. This implies that it is the hole carriers that are collected by the metal electrodes, as opposed to the previous results on various semiconducting devices with p-type bending near the electrode^27–30^. This is also in contrast to the bare STO devices, which exhibit p-type SPCM signals, as shown in Fig. 2. We also note that, thermoelectric currents were also appeared with the opposite polarity with respect to *I*_SPC_, when measured in an ambient condition (Supplementary Informations S4 and S5).

There are two possible explanations for the polarity of the UV-induced SPCM signals in a 2DEG system. The first is the occurrence of n-type electronic band bending near the metal electrodes, in which the potential energy of the electronic band structure decreases towards the middle of the conducting channel. This is a configuration where the hole carriers drift due to the strong electric field formed near the metal contacts following charge separation. Alternatively, the n-type polarity may be attributable to the diffusion of hole carriers towards nearby electrodes when the field-driven effects are negligible^33^, provided that the electron carriers have been removed by, for instance, being trapped at the surface of the oxide layer. However, this is improbable because the Al_2_O_3_ surface is known to capture the hole carriers instead. In the following results, we found that the photocurrent polarity can be successfully interpreted in terms of a new band alignment picture, in which the hole carriers in the valence band are collected by the electrodes while the electrons in the conduction band are blocked by the high potential barrier of the Al_2_O_3_ layer^34, 35^.

### Gate-dependent SPCM signals in the 2DEG device

**Figure 4:**
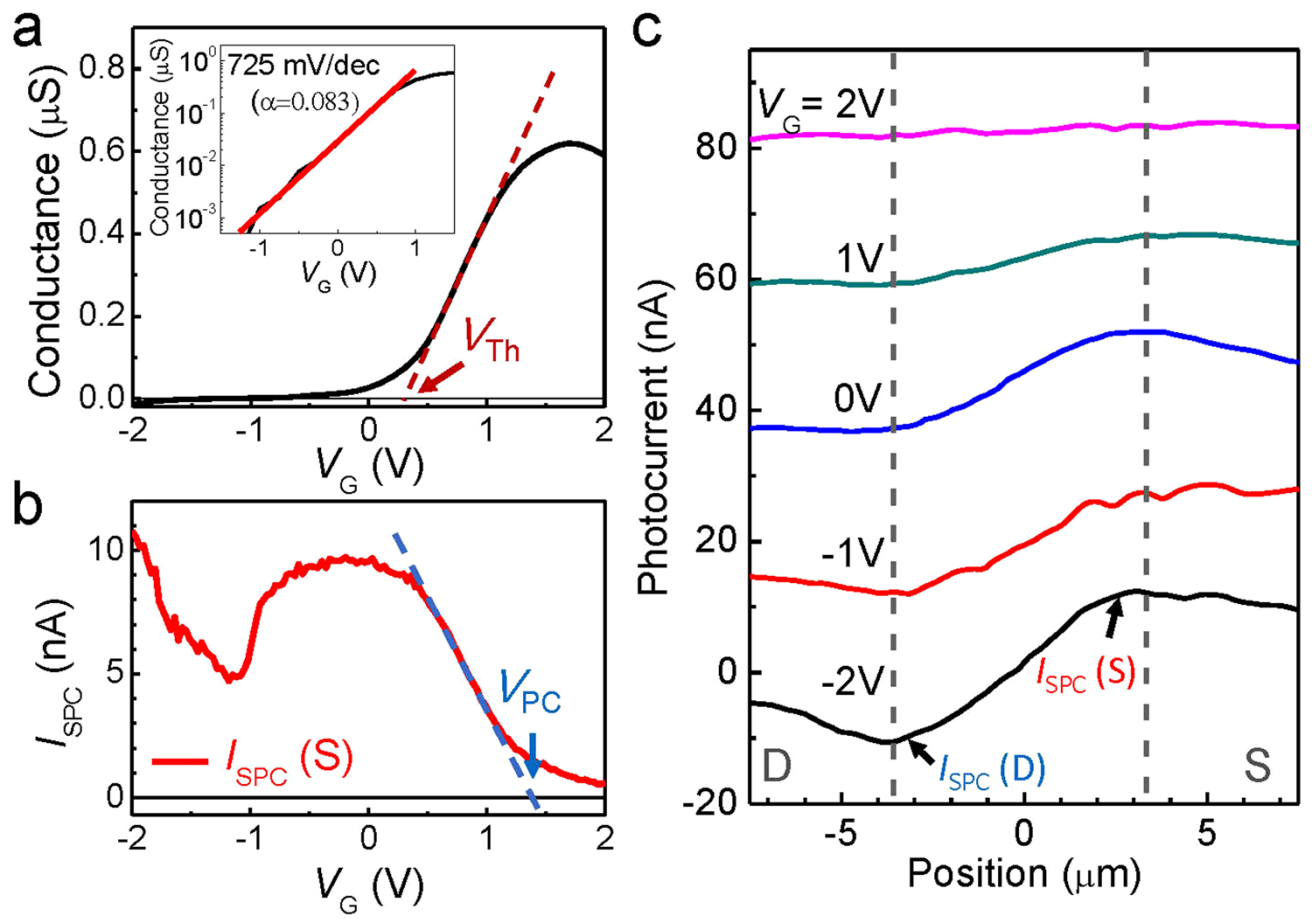
(**a**) Conductance of a 2DEG device as a function of *V*_G_. *V*_Th_ denotes the threshold voltage where the conductance is turned on. (inset) Logarithmic plot of conductance vs *V*_G_ (black line). Red line is a fit to the data. (**b**) *I*_SPC_ as a function *V*_G_ near source electrode. *V*_PC_ denotes the voltage where the photocurrent (*I*_SPC_) is turn-off. (**c**) Photocurrent profile as a function of position along the device channel for various gate bias. The curves are offset for clarity.

In order to investigate the electronic band alignment at the interfaces, the gate-response was studied in conjunction with the SPCM signals obtained near the metal contacts. The gate-response has been studied in an electrolyte configuration using an ionic liquid, as mentioned previously. Recently, electrolyte gating of the 2DEG conduction has been demonstrated in the LAO/STO system^36^, but has not been demonstrated for the Al_2_O_3_/STO system. Figure 4a shows the 2DEG conductance as a function of *V*_G_. Here we varied *V*_G_ very slowly, with steps of 50 mV, waiting 30 s before we obtained each *I–V*_SD_ curve and we plotted the slope of the *I*-*V*_SD_ curve as a function of *V*_G_ (Supplementary Information S6). The sample demonstrates good switching behavior with the turn-on voltage *V*_Th_ at ~0.28 V. The inset shows the logarithmic plot of the gate response, from which we can obtain the subthreshold voltage swing values^27, 37^. Fitting the data to the red line, we found a subthreshold voltage swing of 725 mV/dec, yielding a gate efficiency *α* of 0.083. The relatively low gate efficiency is attributable to the fact that the ionic liquid is not in a direct contact with the conducting channel. This information is very useful for quantifying the voltage offset in the band alignment, as will be shown later. Although we focus on the electrolyte gate response here, interesting memory effects have been observed when we used a back-gate configuration, in which we deposited a gold metal film on the bottom of the STO crystal film (Supplementary Information S7).

The one of *I*_SPC_ signals is shown as functions of *V*_G_ in Fig. 4b for the source electrode. We found that *I*_SPC_ varies significantly with increasing gate bias. We also note that the SPCM signals were not influenced by dynamical changes in *V*_G_, such as the direction and speed of its sweep. At a large negative bias of *V*_G_, the polarity of *I*_SPC_ represents the n-type band bending signals, i.e., positive current near the source electrodes as mentioned previously. The signals decreased gradually as we increased *V*_G_, until they were almost turned off at *V*_G_ = *V*_PC_ ~ 1.38 V. In other words, the SPCM signals decrease and are eventually switched off with increasing *V*_G_, whereas the conductance increases along with *V*_G_ (for *V*_G_ > *V*_Th_). This is a very intriguing event that was not observed in previous SPCM studies on various nanoscale devices including semiconducting NWs, graphenes, and SWNTs^27–30^. In such devices, the gate-dependent SPCM signals tend to increase or decrease along with the device conductance and there is a voltage offset between their switching behaviors.

As shown in Fig. 4c, photocurrent profiles measured along the conduction channel are consistent with those in Fig. 4b. The *I*_SPC_ signals are pronounced at large negative *V*_G_ values, with polarity comparable to the n-type behaviors, whereas they decrease with increasing *V*_G_ until they finally vanish for *V*_G_ > *V*_PC_. We observed similar switching behaviors in the SPCM signals of more than 10 devices, although detailed behaviors depends on the devices. We believe that this is because of the ALD processes in which we deposit the amorphous Al_2_O_3_ layer.

### Band alignment at the STO/Al_2_O_3_ interface

**Figure 5:**
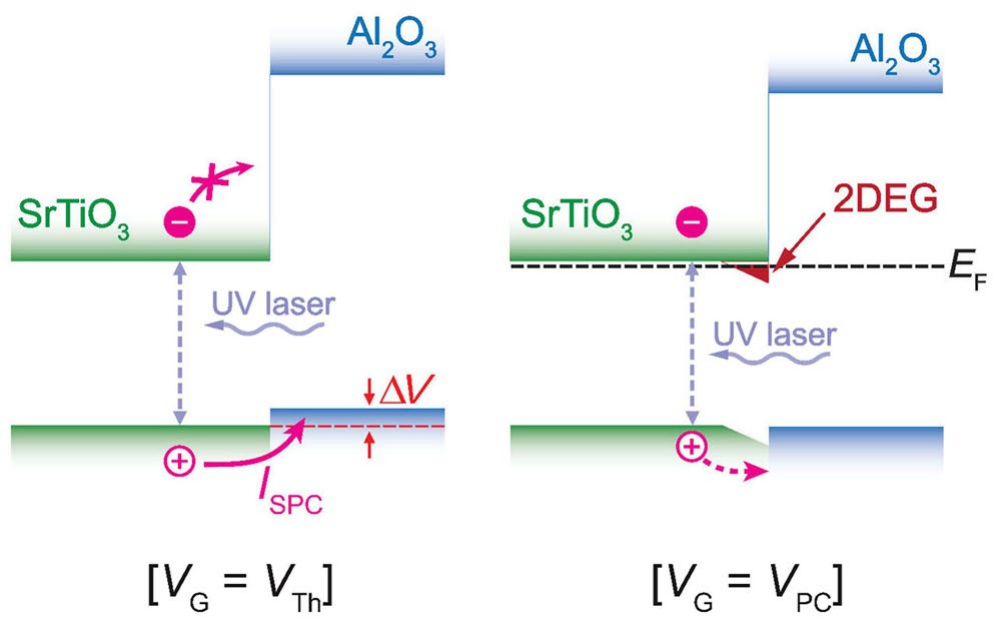
Illustration of a band alignment for Al_2_O_3_/STO oxides heterostructure for *V*_G_ = *V*_Th_ (left) and *V*_G_ = *V*_PC_ (right).

The unique switching behaviors of the 2DEG systems can be interpreted through a novel electronic band alignment schematically illustrated in Fig. 5. From our results, we are forced to conclude that the potential barrier of the valence bands at the interface between the STO and Al_2_O_3_ region is relatively small, whereas there is a large potential barrier for the conduction bands. Similar band diagram has been proposed recently by the density functional calculations^7^ and by the hard x-ray photoelectron spectroscopy^35^, but it has not been addressed in the 2DEG system formed by amorphous Al_2_O_3_ layer. In this configuration, the photogenerated electrons cannot easily penetrate into the metal electrodes due to the large potential barriers. Conversely, the photogenerated holes can easily migrate toward the metal electrodes, resulting in n-type photocurrent signals (i.e., positive *I*_SPC_ for the source electrodes).

The gate-dependent switching behavior of the SPCM signals, which deviates completely from that of the conductance, can also be explained by our band-alignment picture. The increase in conductance for *V*_G_ > *V*_Th_ implies that the formation of the 2DEG is strongly confined near the surface, which is associated with the localized potential well (depicted by the red area). The electron carrier density and, hence, the conductance will increase as the well depth increases with *V*_G_. Conversely, the hole carriers will flow toward the electrode efficiently only for *V*_G_ < *V*_PC_. This flow decreases as *V*_G_ increases, and is finally turned off at *V*_G_ = *V*_PC_. Here, we assume that the gate-dependent valance band offset (between the STO and Al_2_O_3_ layers) determines the amount of the hole migration toward the metal electrode, whereas the band-bending in the depletion layer (associated with the formation of 2DEG) has a negligible influence on the SPCM signals.

Finally, from the observed gate efficiency values and the offset voltage Δ*V*_G_ = |*V*_PC_ − *V*_Th_|, we quantified the potential barrier height of the conduction band, as depicted by Δ*V* in Fig. 5. This corresponds to a valence band offset between the STO and Al_2_O_3_ for *V*_G_ = *V*_Th_. The band alignment information is crucial for understanding and optimizing the oxide materials, and has not yet been reported. For the device shown in Fig. 4, Δ*V*_G_ is 1.10 V. From the subthreshold voltage swing value and the relation Δ*V* = *α*Δ*V*_G_, the valence band offset was found at 91.03 meV. In the 9 devices we tested, Δ*V* reached 85 meV on average (with a standard deviation of 18 meV) (Supplementary Information S8). This is a small value as compared to the valence band offset (~600 meV) reported at the spinel/perovskite γ-Al_2_O_3_/STO heterointerface obtained from the hard x-ray photoelectron spectroscopy^35^, which is likely because we used amorphous Al_2_O_3_ film. Band alignments in the complex oxide systems have been largely unexplored, although this information plays a crucial role for optimizing the device performance. For instance, we will be able to visualize the change in the band alignments as we attempt to lower the electron barrier height using various interface engineering techniques. Therefore, our approach (SPCM incorporated with UV light source) will provide a powerful tool for characterizing various localized information for nanoscale devices that include oxide materials with a large bandgap, especially when their electronic band information is not easily accessible using conventional transport measurements.

## Conclusion

In conclusion, using an SPCM coupled with a UV laser source, we studied the electronic band alignment at an Al_2_O_3_/STO heterointerface forming a 2DEG layer. The 2DEG device shows n-type behavior with clear conductance switching behavior (at *V*_G_ = *V*_Th_) under an ionic liquid gating environment, whereas there exists a tunneling current through the oxide layer. The energy of the UV laser source was greater than the bandgap of the STO layer, enabling carrier generation that contributes to the photocurrent through migration towards the metal electrode. The polarity of the localized photocurrent signals at the 2DEG device exhibited n-type responses, indicating that the UV-induced hole carriers were collected by the metal electrodes. The SPCM signals decreased with increasing gate bias until they were finally turned off at *V*_G_ = *V*_PC_, whereas the DC conductance increased with *V*_G_ for *V*_G_ >  *V*_Th_. On the other hand, the SPCM of the bare STO device (without the Al_2_O_3_ layer) demonstrates typical p-type behavior with gate-dependent polarity switching behavior, as observed in other conventional semiconducting devices. Finally, we were able to determine the electronic band alignment in the 2DEG devices and obtain the valence band offset between the STO and Al_2_O_3_ layers, which was 85 meV on average at *V*_G_ = *V*_Th_. Our work will form the basis for further study into the various localized electronic states in oxide electronic devices in order to optimize their performance.

## Methods

### Experimental setup details

Diode-pumped solid state (DPSS) laser at 355 nm (Cobolt) was focused by a water-immersion objective lens (Olympus Corporation; 60X, NA 0.9) and raster-scanned using galvanometer scanning mirrors (Thorlabs, Inc.). The laser amplitude was modulated by a photoelastic modulator (PEM) at 120 kHz, enabling us to achieve both rapid scanning and improved signal-to-noise ratio (SNR) simultaneously. The photocurrent signals were measured using a fast current pre-amplifier (Femto Messtechnik GmbH) and a lock-in amplifier (AMETEK, Inc.). Each SPCM image is taken for ~5 s. For efficient gating, we used the electrolyte gating method with a Pt electrode submerged in ionic liquid (1-Ethyl-3-methylimidazolium bis(trifluoromethylsulfonyl)imide, Merck)^31^. We used a Polydimethylsiloxane (PDMS) wall to confine the liquid near the objective lens.

### Fabrication of Al_2_O_3_/SrTiO_3_ devices

We fabricated FET devices with a 2DEG layer formed at the Al_2_O_3_/STO interface. The amorphous Al_2_O_3_ films were grown on a TiO_2_-terminated (001) STO crystalline substrate (purchased from MTI Corporation) at 300 °C using the ALD technique. Here, TiO_2_-termination was achieved through chemical etching^38, 39^. Trimethylaluminum (TMA, Al(CH_3_)_3_) was used as the Al-precursor and H_2_O was used as the oxygen source for the deposition of the Al_2_O_3_ films^20, 23^. The metal electrodes were defined using conventional photolithography, followed by metal evaporation (Ti/Pt).

## Electronic supplementary material

Supplementary Information

## References

[1] Reyren N (2007). Superconducting interfaces between insulating oxides. Science.

[2] Lee JS (2013). Titanium dxy ferromagnetism at the LaAlO_3_/SrTiO_3_ interface. Nature Materials.

[3] Ohtomo A, Hwang HY (2004). A high-mobility electron gas at the LaAlO_3_/SrTiO_3_ heterointerface. Nature.

[4] Thiel S, Hammerl G, Schmehl A, Schneider CW, Mannhart J (2006). Tunable quasi-two-dimensional electron gases in oxide heterostructures. Science.

[5] Zubko P, Gariglio S, Gabay M, Ghosez P, Triscone JM (2011). Interface physics in complex oxide heterostructures. Annual Review of Condensed Matter Physics.

[6] Mannhart J, Schlom DG (2010). Oxide Interfaces-An opportunity for electronics. Science.

[7] Bjaalie L, Himmetoglu B, Weston L, Janotti A, Van De Walle CG (2014). Oxide interfaces for novel electronic applications. New J. Phys..

[8] Förg B, Richter C, Mannhart J (2012). Field-effect devices utilizing LaAlO_3_-SrTiO_3_ interfaces. Appl. Phy. Lett.

[9] Kim, S. K. *et al*. Electric-field-induced shift in the threshold voltage in LaAlO_3_/SrTiO_3_ heterostructures. *Sci*. *Rep*. **5** (2015). 10.1038/srep08023 PMC4306114 25620684

[10] Christensen DV (2016). Electric field control of the γ-Al_2_O_3_/SrTiO_3_ interface conductivity at room temperature. Appl. Phys. Lett..

[11] Christensen DV (2013). Controlling interfacial states in amorphous/crystalline LaAlO_3_/SrTiO_3_ heterostructures by electric fields. Appl. Phys. Lett..

[12] Au K, Li DF, Chan NY, Dai JY (2012). Polar liquid molecule induced transport property modulation at LaAlO_3_/SrTiO_3_ heterointerface. Adv. Mater..

[13] Chan NY (2014). Highly Sensitive Gas Sensor by the LaAlO_3_/SrTiO_3_ Heterostructure with Pd Nanoparticle Surface Modulation. Adv. Mater..

[14] Xie Y, Hikita Y, Bell C, Hwang HY (2011). Control of electronic conduction at an oxide heterointerface using surface polar adsorbates. Nat. Commun..

[15] Pentcheva R, Pickett WE (2009). Avoiding the polarization catastrophe in LaAlO_3_ overlayers on SrTiO_3_(001) through polar distortion. Phys. Rev. Lett..

[16] Hwang HY (2006). Tuning interface states. Science.

[17] Hwang HY (2012). Emergent phenomena at oxide interfaces. Nat. Mater..

[18] Nakagawa N, Hwang HY, Muller DA (2006). Why some interfaces cannot be sharp. Nature Mater.

[19] Chen YZ (2013). A high-mobility two-dimensional electron gas at the spinel/perovskite interface of γ-Al_2_O_3_/SrTiO_3_. Nat. Commun..

[20] Lee SW, Liu Y, Heo J, Gordon RG (2012). Creation and Control of Two-Dimensional Electron Gas Using Al-Based Amorphous Oxides/SrTiO_3_ Heterostructures Grown by Atomic Layer Deposition. Nano Lett..

[21] Chen Y (2011). Metallic and insulating interfaces of amorphous SrTiO_3_-based oxide heterostructures. Nano Lett..

[22] Liu ZQ (2013). Origin of the Two-Dimensional Electron Gas at LaAlO_3_/SrTiO_3_ Interfaces: The Role of Oxygen Vacancies and Electronic Reconstruction. Phys. Rev. X.

[23] Lee SW, Heo J, Gordon RG (2013). Origin of the self-limited electron densities at Al_2_O_3_/SrTiO_3_ heterostructures grown by atomic layer deposition-oxygen diffusion model. Nanoscale.

[24] Kormondy KJ (2015). Quasi-two-dimensional electron gas at the epitaxial alumina/SrTiO_3_ interface: Control of oxygen vacancies. J. Appl. Phys..

[25] Leskelä M, Ritala M (2003). Atomic Layer Deposition Chemistry: Recent Developments and Future Challenges. Angew. Chem. Int. Ed..

[26] Graham R, Yu D (2013). Scanning photocurrent microscopy in semiconductor nanostructures. Mod. Phys. Lett. B.

[27] Ahn Y, Dunning J, Park J (2005). Scanning photocurrent imaging and electronic band studies in silicon nanowire field effect transistors. Nano Lett..

[28] Ahn YH, Tsen AW, Kim B, Park YW, Park J (2007). Photocurrent imaging of p-n junctions in ambipolar carbon nanotube transistors. Nano Lett..

[29] Park JK, Son BH, Park JY, Lee S, Ahn YH (2013). High-speed scanning photocurrent imaging techniques on nanoscale devices. Curr. Appl. Phys..

[30] Son BH (2014). Imaging Ultrafast Carrier Transport in Nanoscale Field-Effect Transistors. ACS Nano.

[31] Ye J (2011). Accessing the transport properties of graphene and its multilayers at high carrier density. Proc. Natl. Acad. Sci. USA.

[32] Park JK, Son BH, Park JY, Lee S, Ahn YH (2014). Imaging surface charge distribution near carbon nanotube device in aqueous environments. Appl. Phys. Lett..

[33] Park JK (2012). Diffusion length in nanoporous photoelectrodes of dye-sensitized solar cells under operating conditions measured by photocurrent microscopy. J. Phys. Chem. Lett..

[34] Cen C, Thiel S, Mannhart J, Levy J (2009). Oxide nanoelectronics on demand. Science.

[35] Schütz P (2015). Band bending and alignment at the spinel/perovskite γ-Al_2_O_3_/SrTiO_3_ heterointerface. Phys. Rev. B.

[36] Zeng S (2016). Liquid-Gated High Mobility and Quantum Oscillation of the Two-Dimensional Electron Gas at an Oxide Interface. ACS Nano.

[37] Rosenblatt S (2002). High Performance Electrolyte Gated Carbon Nanotube Transistors. Nano Lett..

[38] Kawasaki M (1994). Atomic control of the SrTiO_3_ crystal surface. Science.

[39] Koster G, Kropman BL, Rijnders GJHM, Blank DHA, Rogalla H (1998). Quasi-ideal strontium titanate crystal surfaces through formation of strontium hydroxide. Appl. Phys. Lett..

