# Supplementary material for: Electronic Band Alignment at Complex Oxide Interfaces Measured by Scanning Photocurrent Microscopy
Source: Sci Rep. 2017 Jun 19;7:3824. doi: 10.1038/s41598-017-04265-9 (PMC5476647; doi:10.1038/s41598-017-04265-9)
Supplement: Supplementary file 1 — Supplementary Information [file 41598_2017_4265_MOESM1_ESM.pdf]

Supplementary Information

Electronic Band Alignment at Complex Oxide Interfaces Measured by Scanning Photocurrent Microscopy

*J. H. Yoon, H. J. Jung, J. T. Hong, Ji-Yong Park, Soonil Lee, S. W. Lee, and Y. H. Ahn**

Department of Physics and Department of Energy Systems Research, Ajou University, Suwon 16499, Korea

*Corresponding author. Electronic mail: ahny@ajou.ac.kr

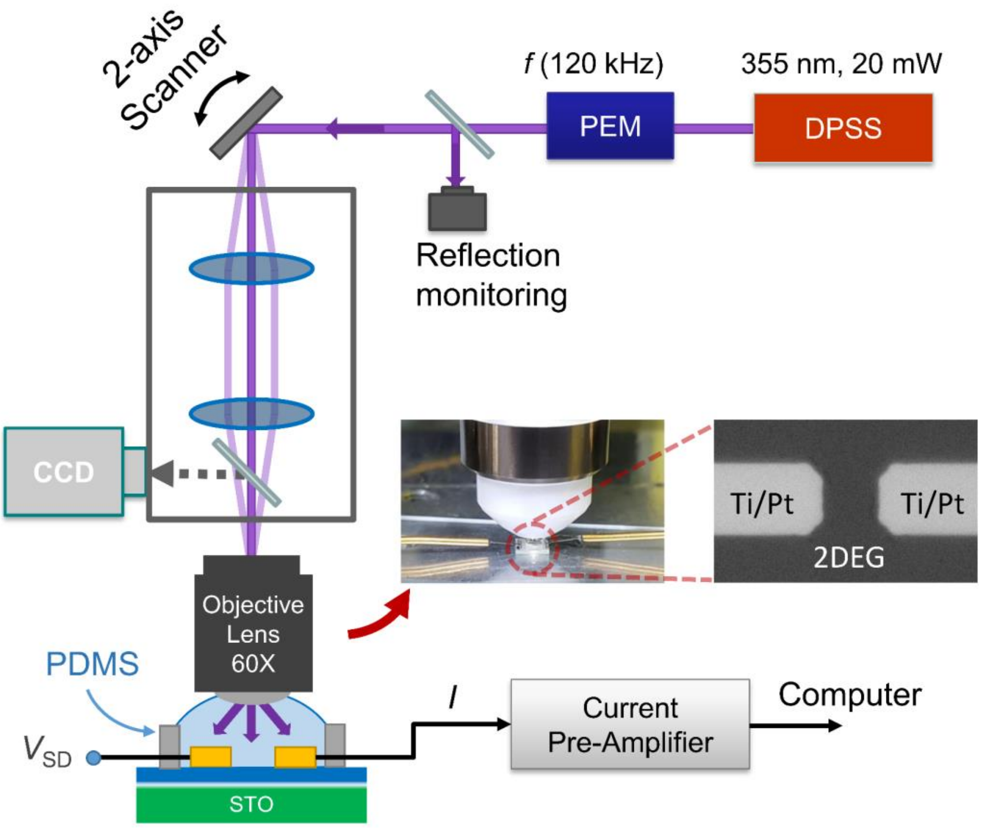

Figure S1. Experimental setup details Diode-pumped solid state (DPSS) laser at 355 nm (Cobolt) was focused by a water-immersion objective lens (Olympus Corporation; 60X, NA 0.9) and raster-scanned using galvanometer scanning mirrors (Thorlabs, Inc.). The laser amplitude was modulated by a photoelastic modulator (PEM) at 120 kHz, enabling us to achieve both rapid scanning and improved signal-to-noise ratio (SNR) simultaneously. The photocurrent signals were measured using a fast current pre-amplifier (Femto Messtechnik GmbH) and a lock-in amplifier (AMETEK, Inc.). Each SPCM image is taken for ~ 5 s. A Pt wire submerged in the ionic liquid solution works as a working electrode for the electrolyte gating. We used a Polydimethylsiloxane (PDMS) wall to confine the liquid near the objective lens.

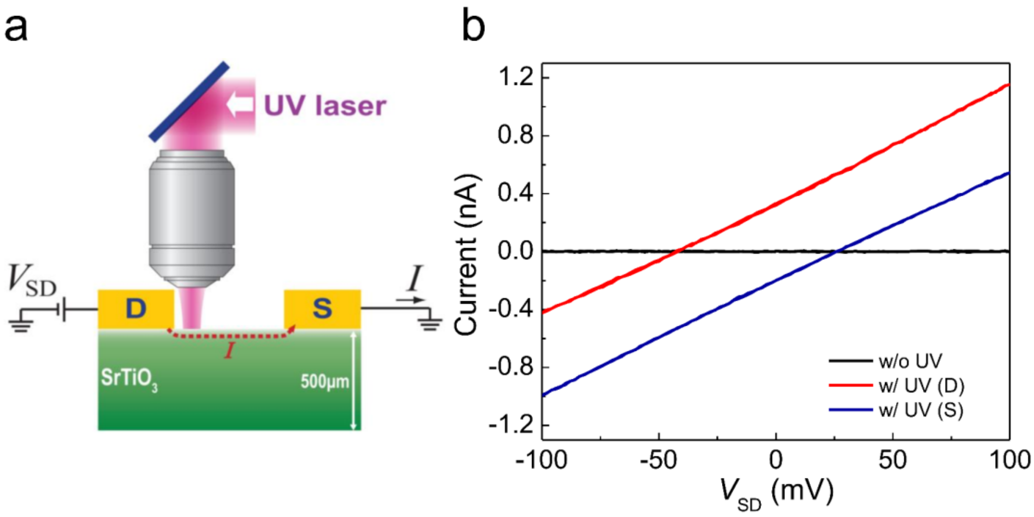

Figure S2. UV light response of bare STO device (a) Schematic illustration SPCM measurements on a bare STO device. (b) I-V_{SD} curve of the STO device in an ambient condition without the illumination of the UV laser (black line). Shown together as a red (blue) line is I-V_{SD} curve when the UV illuminate near drain (source) electrode. We could not observe the device conductance without the UV light, whereas the conductance of the device appears with the UV light illumination. It is surprising that the conduction channel is formed even if we locally illuminate the STO device (with a spot size of ~300 nm), which is likely due to the high mobility and the large diffusion length of STO.

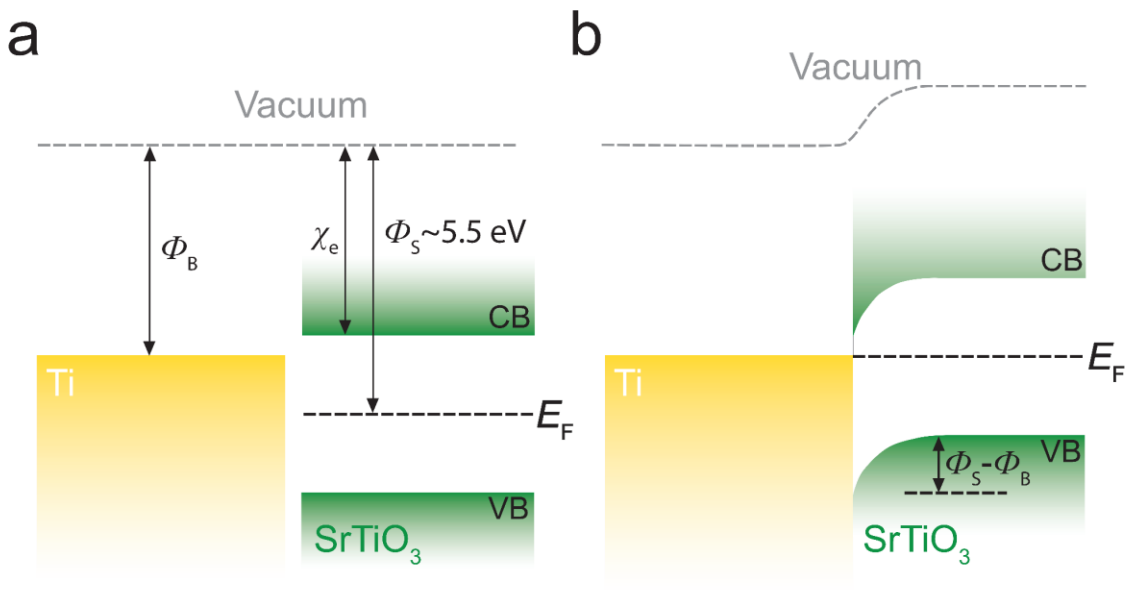

Figure S3. Estimation of STO/metal band alignment. (a) Illustration of a band alignment of Ti metal electrode and STO layers before the formation of junction (b) Band alignment at the metal/STO contact in the thermal equilibrium condition. The band alignment implies the formation of the p-type electronic band-bending near the metal contacts, which is consistent with the results shown in the main text.

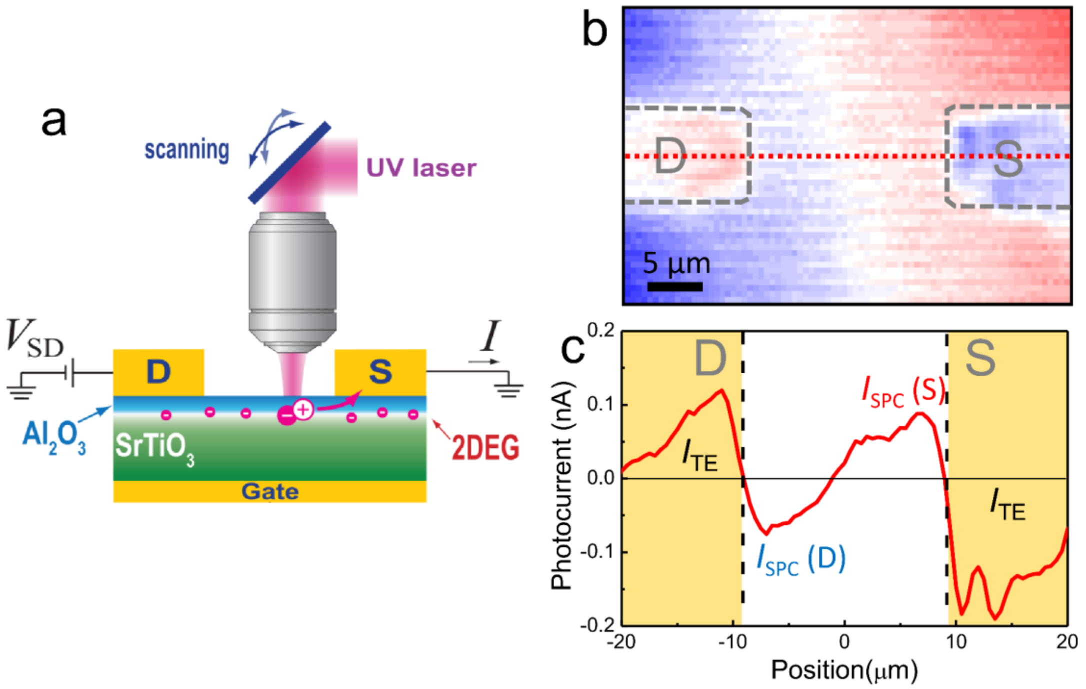

Figure S4. SPCM Measurements on 2DEG device in ambient condition. (a) Schematics of SPCM measurements in an ambient condition. (b) A SPCM image of 2DEG device with a channel length of 20 μm measured in ambient condition. (c) Photocurrent profile as a function of position extracted from (b) along the red dotted line. The thermoelectric currents (I_{TE}) are clearly distinguished from those at the 2DEG region (I_{SPC}). When the metal electrodes are illuminated, strong SPCM signal (I_{TE}) appears with p-type polarity (i.e., positive current for drain electrode), whereas the SPCM on 2DEG region near the metal contact (I_{SPC}) shows typical n-type polarity as shown in the main text for the liquid-gate environments. The signal on the metal electrodes is attributable to the thermoelectric effects, which originates from the laser induced heating of the electrodes. It is likely that this effect is suppressed in the ionic liquid gate environments as shown in the main text.

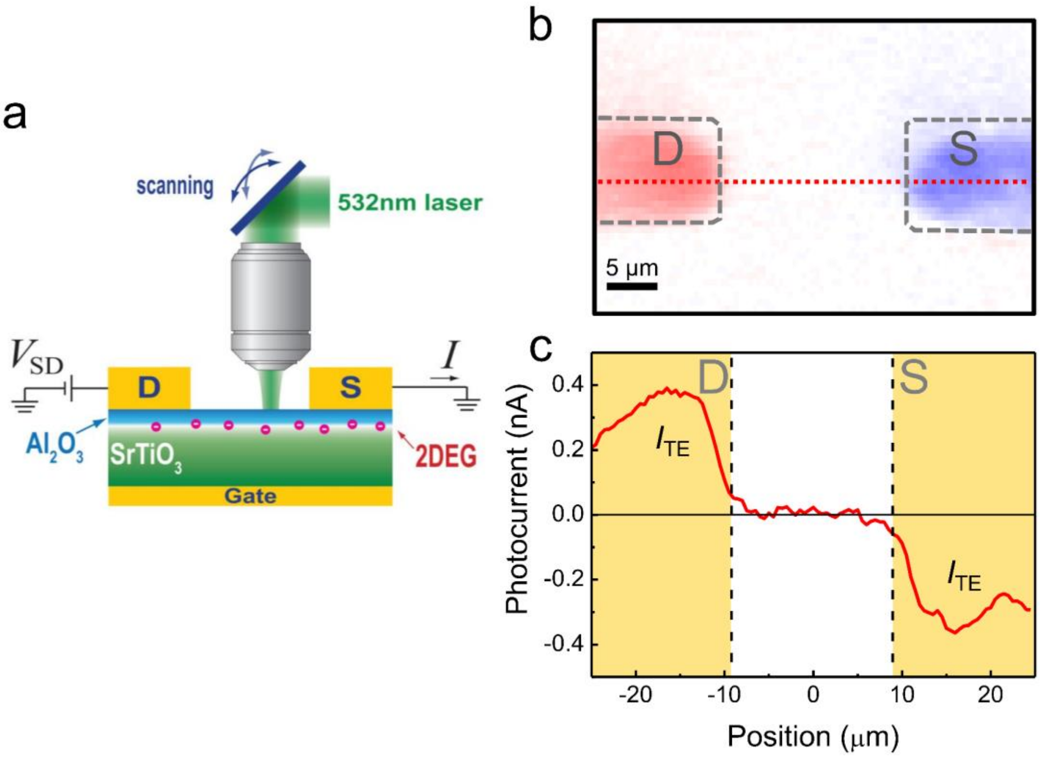

Figure S5. SPCM Measurements on 2DEG device with 532 nm laser. (a) Schematics of SPCM measurements in an ambient condition with a green laser (532 nm). (b) A SPCM image of 2DEG device measured in ambient condition on the same device shown in Fig. S5. (c) Photocurrent profile as a function of position extracted from (b) along the red dotted line. Strong SPCM signal appears on the metal electrodes with p-type polarity, whereas no noticeable I_{SPC} has been observed the 2DEG region is illuminated, including the region near the metal contacts. In other words, only thermoelectric effect was observed with the green laser illumination. This is because we could not induce the carrier generation in the 2DEG region, whereas metal electrode can be heated by the green laser.

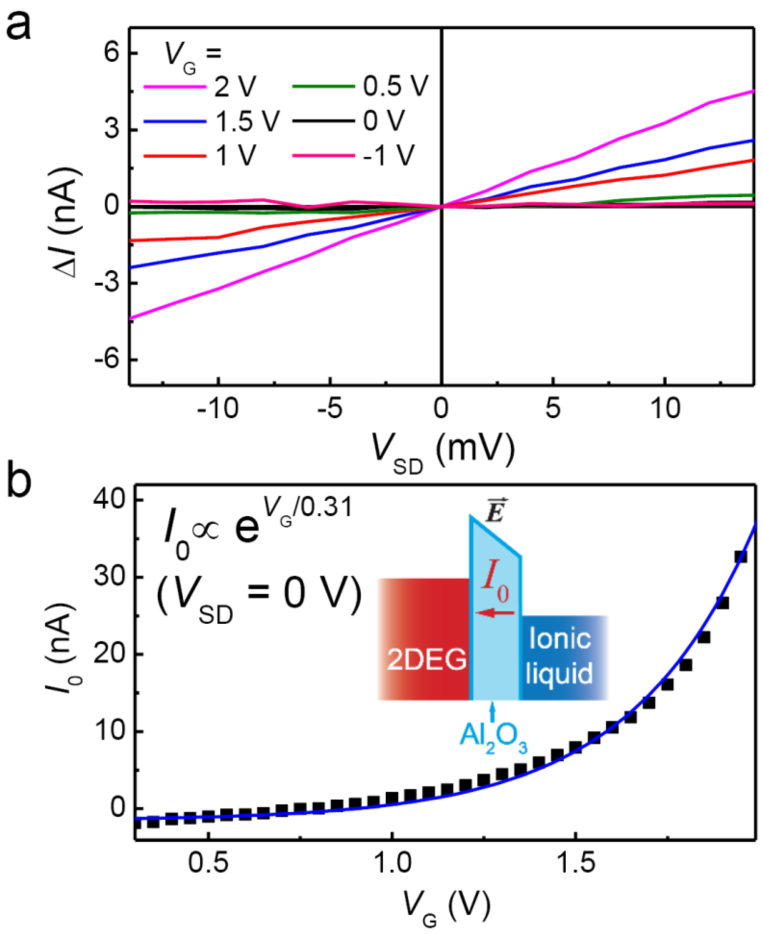

Figure S6. DC conductance of 2DEG device as a function of electrolyte gate bias. (a) Plot of $\Delta I = I - I_0$ as a function of V_{SD}, for different V_G's, where I_0 is the current measured at V_{SD} = 0 V (i.e., y-intercept in I-V_G curve). Here we varied V_G very slowly, with steps of 50 mV, waiting 30 s before we obtained each I–V_{SD} curve. The slope of the I-V_{SD} curve changed dramatically as a function of V_G. The device shows clear switching behavior as the conductance increases with V_G and it is virtually turned off for $V_G < 0$ V, as shown in Figure 4 of the main text. (b) The current (I_0) as a function of V_G (black square dot) at V_{SD} = 0. Shown together as blue solid line is a fitting curve. The curve was fitted by the relation $I_0 \propto \mathrm{Exp}(V_G/V_\theta)$, and we obtained a V_θ of 0.31 V (solid line). This strongly indicates that I_0 corresponds to the tunneling current through the Al_2O_3 layer sandwiched between the electrolyte and 2DEG layer. (c)

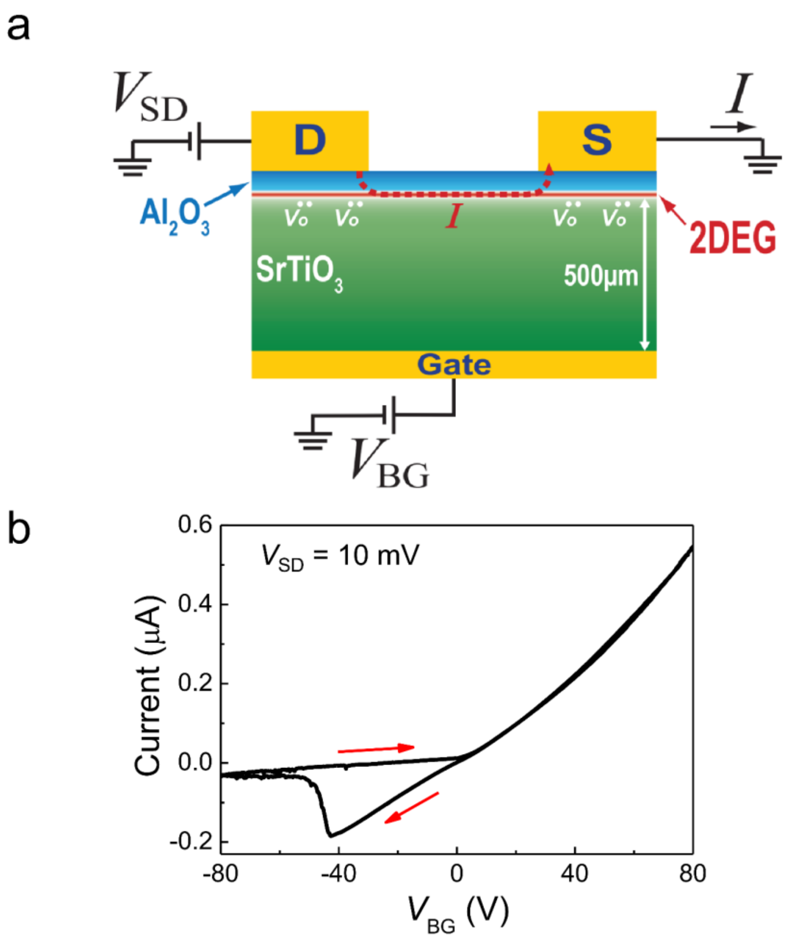

Figure S7. DC conductance of 2DEG device with back-gate geometry. (a) Schematic of the device geometry with a back-gate electrode. The gold film were deposited on the back side of the STO substrate (with the thickness of 500 μm). (b) I-V_{BG} curve for a fixed V_{SD} = 10 mV show interesting behaviors in a negative bias region, depending on the sweep direction. In other words, the current level decreased to −185 nA and suddenly increases to −30 nA for $V_{BG} < -40$ V. The hump in I-V_{BG} curve indicates an abrupt increase in the resistance of the device for $V_{BG} < -40$ V. Similar memory effects have been observed in LAO/STO device with the top gate geometry[1]; however it has not been reported in an Al_2O_3/STO system. This effect can been attributed to the presence of the deep trap levels or ionic movements induced by a high electric fields.

V_{Th} (V)	V_{PC} (V)	α	ΔV (meV)
0.28	1.38	0.083	91.03
-0.24	1.06	0.056	72.61
0.73	2.48	0.070	122.67
0.42	1.76	0.072	97.26
0.60	1.48	0.105	91.75
0.64	1.62	0.078	76.66
0.26	1.46	0.063	75.26
0.57	1.72	0.055	64.12
0.44	0.84	0.180	73.22
		**Average**	84.94 meV
		**Standard deviation**	17.82 meV

Figure S8. Statistics for barrier height measurements. Series of results from 9 different 2DEG devices for the measurement of barrier height ΔV.

References

(1) Kim, S. K.; Kim, S. I.; Lim, H.; Jeong, D. S.; Kwon, B.; Baek, S. H.; Kim, J. S. Electric-field-induced shift in the threshold voltage in $LaAlO_3/SrTiO_3$ heterostructures. *Sci. Rep.* **5**, 8023 (2015).
